# Experimental Study on Thermosensitive Hydrogel Used to Extinguish Class A Fire

**DOI:** 10.3390/polym13030367

**Published:** 2021-01-24

**Authors:** Li Ma, Xiao Huang, Youjie Sheng, Xixi Liu, Gaoming Wei

**Affiliations:** 1 College of Safety Science and Engineering, Xi’an University of Science and Technology, Xi’an 710054, China; serendipity_hx@163.com (X.H.); youjies@xust.edu.cn (Y.S.); xi_liuliu@163.com (X.L.); 20180326@163.com (G.W.); 2 Shaanxi Key Laboratory of Prevention and Control of Coal Fire, Xi’an 710054, China

**Keywords:** extinguishing agent, thermosensitive hydrogel, class A fire, viscosity

## Abstract

Hydrogels are crosslinked polymers that become fully swollen when placed in aqueous environments. They are widely used in the field of firefighting because they can remarkably increase the viscosity and wettability of water. In this study, a thermosensitive hydrogel used to effectively suppress class A fire was synthesized by using methylcellulose, sodium polyacrylate, and magnesium chloride. The structure, surface activity and viscosity of the hydrogel were characterized. Fire extinguishing performance was evaluated based on small-scale and large-scale experiments. The results showed that a phase transition of the hydrogel occurred when the temperature rose from 50 °C to 80 °C. After the phase transition, the hydrogel showed a higher viscosity and lower surface tension, which was conducive to attach to the surface of the burning material and acting as an effective barrier to isolate oxygen. The small-scale fire extinguishing tests indicated that the concentration of the hydrogel solution has an eminent influence on fire extinguishing performance. The optimum concentration for extinguishing performance was around 6 wt%. The large-scale experiments demonstrated that the fire-extinguishing performance of this thermosensitive hydrogel was superior to the two other commercial water-based fire extinguishing agents, as it prevented re-ignition highly efficiently.

## 1. Introduction

Fires often cause extensive wealth loss and pose a momentous threat to human life. Fire extinguishing agents with high-efficient combustion suppression are crucial to prevent fire hazards [1,2,3]. Water is used to cool flames because of its high heat capacity [4], but the strong fluidity of water will cause it to be wasted due to the massive loss in most fire-fighting operations, and improper disposal of fire wastewater can easily lead to water pollution [5,6]. Therefore, it is an urgent task to research and develop a new environmentally friendly fire extinguishing agent.

Hydrogels, as high-molecular-weight polymers, have characteristics capable of absorbing a large quantity of liquid. They have been widely used in fire prevention and extinguishing during the past few decades [7,8,9]. However, the traditional hydrogels have the inconvenience of storage, transportation, blockage of pipelines, and difficult disposal of fire extinguishing residues due to their high viscosity at room temperature [10,11,12,13]. Recently, thermosensitive hydrogels have attracted widespread attention for their unique temperature responses, which can solve the above defects of traditional hydrogels. Thermosensitive hydrogels, a type of functional polymer material with wide applications, have an abrupt change in phase behavior when the temperature reaches a specific value [14,15]. In addition, the special temperature value is often called the lower critical solution temperature (LCST) in some previous studies. Thermosensitive hydrogels exist in liquid form below the LCST and solidify into a gel after the temperature exceeds the LCST [16,17]. The temperature sensitivity is associated with the role of the hydrophilic and hydrophobic groups in its structure. It has strong fluidity and low viscosity at room temperature, and can rapidly release water at a high temperature and form glue on the surface of the burning material [18]. Currently, thermosensitive hydrogels have been extensively studied in the fields of medicine, agriculture, etc. [19,20,21].

However, so far research regarding thermosensitive hydrogels in the field of fire-fighting were considerably rare. The previous research studies primarily focused on the thermal sensitivity, flame retardancy, and inhibitory effects on coals of thermosensitive hydrogels. Therefore, there is still a huge research gap to be addressed in the fire-fighting field. Deng et al. [22] prepared a linear copolymer P (NIPA-co-SA) hydrogel and established the numerical relationship between the monomer ratio and the critical transition temperature (LCST); Hu et al. [23] prepared a thermosensitive hydrogel by free radical polymerization, and found the volume phase transition temperature of the hydrogel decreased at a higher N-isopropylacrylamide amount; Yu et al. [24] used hydrogel and natural cotton fabric to prepare a new type of fireproof material, which can achieve ideal fireproof performance; Tsai et al. [25] prepared a thermosensitive hydrogel (P(NIPA-co-SA)), and the experimental results showed that the gel has a remarkably inhibitory effect on the spontaneous combustion of anthracite and coking coal; Jiang and Dou [26] prepared a polyacrylic acid–methacrylamide hydrogel grafted with chitosan (CTS-gP(AA-co-MAA)) by aqueous solution polymerization, and experiments proved it can substantially inhibit the initial oxidation of coal. It is unclear whether the thermosensitive hydrogels can extinguish class A fire effectively or not. Therefore, more experiments need to be carried out to study the fire extinguishing performance of thermosensitive hydrogels.

With the call for cost reduction and the protection of the environment, the preparation of gels using natural polyhydroxy materials has become a hot research topic. Cellulose-based hydrogel combines the biodegradability and the large availability of cellulose in nature [15,27,28], along with the smart sensitive behavior and the low cost of cellulose derivatives, which have various ranges of applications. In this study, we used methylcellulose, sodium polyacrylate, and magnesium chloride as raw materials to prepare a thermosensitive hydrogel. The structure, surface activity, viscosity, fire extinguishing performance of the hydrogel were characterized. The present study can provide technical support for fire prevention through theoretical analysis and experimental testing.

## 2. Materials and Methods 

### 2.1. Materials

Methylcellulose (MC, molecular weight 40,000~160,000, analytically pure) was purchased from Tianjin Fuchen Chemical Reagent Co., LTD. (Tianjin, China); sodium polyacrylate (PAAS, average molecular weight 50,000, analytically pure) was obtained from Shandong Muheng Material Technology Co., LTD. (Shandong, China) As a special surfactant, PAAS is highly efficient at reducing surface tension. Moreover, it has good heat resistance such as it can form a fire-resistant layer after absorbing water, thereby playing a role in resisting flames [29]; Magnesium chloride (MgCl_2_, analytically pure) was provided by Tianjin Kemiou Chemical Reagent Co., LTD. (Tianjin, China) MgCl_2_ is an effective fire-retardant, and has been widely used for fire retardation treatment of polymers. It had catalytic action on the polymerization of levoglucosan in cellulose at 250 °C and increased the primary char yield of cellulose heated at 400 °C [30,31]; deionized water was produced by a water purification machine in the laboratory.

### 2.2. Gel Preparation

**Figure 1:**
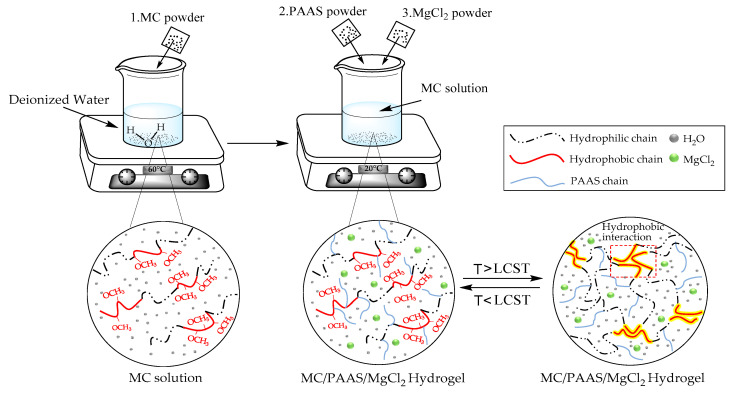
Preparation process of methylcellulose (MC)/ sodium polyacrylate (PAAS)/MgCl_2_ gel.

MC and PAAS powder were put into a vacuum drying oven and dried for 24 h. MC powder was weighed and dissolved in 60 °C water for 2 h to obtain a 1.3 wt% aqueous solution. Similarly, a certain amount of dried PAAS (2 wt%) and MgCl_2_ (6 wt%) powder were added to the solution. The mixture was stirred and dissolved for 2 h to obtain the sample solution. The solution was allowed to remove bubbles after standing at room temperature for 24 h. The hydrogel solutions which were diluted into aqueous solutions with several concentrations (1 wt%, 2 wt%, 3 wt%, 4 wt%, 5 wt%, 6 wt%, 7 wt%, 8 wt%, and 9 wt%) were prepared. The operation process is shown in Figure 1.

### 2.3. Performance Testing

#### 2.3.1. Study on the Structure of the Gel

The structure of the gel was confirmed by the Fourier-transform infrared (FTIR) spectra of a Nicolet iN10 spectrometer (Thermo Fisher Scientific, Waltham, MA, USA) from the wavenumber range of 400 to 4000 cm^−1^.

#### 2.3.2. Surface Tension Test

At 20 °C, the QBZY-3 automatic surface tensiometer (Shanghai Pingxuan Scientific Instrument Co., Ltd, Shanghai, China)was used to test the surface tension of hydrogel solutions with various concentrations. Then ae CL-2 constant temperature water bath was employed to heat up to 80 °C to simulate the high-temperature environment. At this time, the gels’ surface tension after the phase change was tested. The measurement range is 0–110 mN/m, the resolution is less than 0.05 mN/m, the sampling period is 110–200 s, and the maximum power consumption is less than 150 W.

#### 2.3.3. Viscosity Test

The viscosity of gels with different concentrations was tested at 20 and 80 °C by a DV-1 digital viscometer (Beijing Jingmeirui Technology Co., Ltd., Beijing, China). Each condition was repeatedly measured 2–3 times, and the average value was taken as the test results.

### 2.4. Fire Extinguishing Experiment

Fire extinguishing efficiency is mainly determined by fire extinguishing time. Small-scale extinguishing experiments are used to select a thermosensitive hydrogel with better concentration for actual firefighting. Then the better thermosensitive hydrogel is compared with two other extinguishing agents through large-scale experiments, which are commonly used in the market.

**Figure 2:**
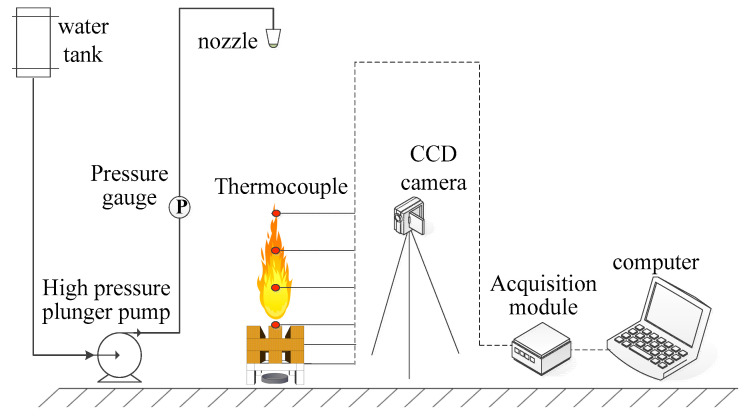
Experimental fire-suppression system.

The small-scale fire-suppression system designed by our research team was an original system. The details of the experimental device are demonstrated in Figure 2. A wood stack (10.5 cm × 15 cm) made of pine wood (10% to 13% moisture content) was used as the burning material. An ignition tray with a diameter of 8.0 cm and a height of 1.5 cm is placed directly under the wood stack; A thermocouple tree containing six thermocouples was set up to measure the flame temperatures. In each experiment, 5 L thermosensitive hydrogel of different concentrations was put into the water tank. The experimental pressure was designed at 6 MPa and the flow rate at 0.4 L/min. An amount of 10 g alcohol in the oil pan was ignited and the wood stack maintained burning for 180 s. The hydrogel was sprayed when the wood stack was pre-burned for 180 s. Each test was repeated three times to achieve average results. The fire-extinguishing process was recorded using a Logitech HD 1080 camera (Logitech (China) Technology Co., Ltd, Shanghai, China).

The large-scale fire platform was designed according to GB 17835-2008. The prepared thermosensitive hydrogel was compared to two other extinguishing agents. One is an ordinary gel extinguishing agent and the other is a foam extinguishing agent. Standard 2A wood stacks (64.0 cm × 63.5 cm) made of pine wood (10% to 13% moisture content) were used as the burning material. An ignition plate with a diameter of 50.0 cm and a height of 20.0 cm was placed under the wood stack. The data log system consisted of a thermocouple tree with eight thermocouples and a camera, recording the temperature changes at different locations and flame behaviors during the fire-extinguishing experiments. The wood stack was ignited and maintained burning for 260 s to achieve a steady state. Then the fire extinguishing agent was sprayed from the top, bottom and side of the wood stack and the distance from the wood stack was no less than 1.8 m. If no re-ignition occurred within 10 min after the open flame was extinguished, it was recorded as a successful fire extinguishing. Each test was repeated three times and the average values were computed. 

## 3. Results and Discussion

### 3.1. Infrared Spectrum Analysis

**Figure 3:**
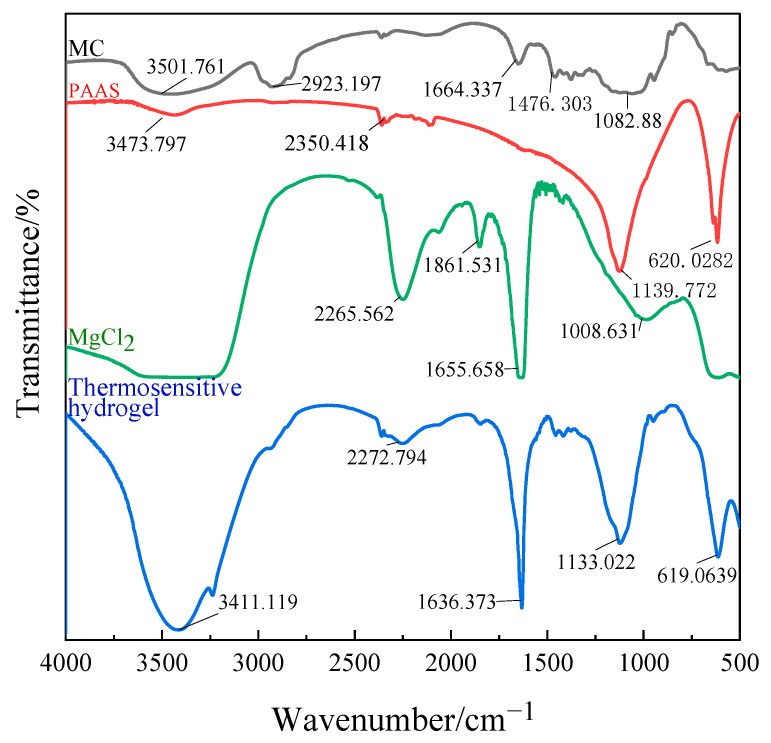
Infrared spectrogram of the thermosensitive hydrogel.

To confirm the structure of the gel, the FTIR spectra of MC, PAAS, MgCl_2_, and the thermosensitive hydrogel are presented in Figure 3. 

A broad spectrum can be observed in the range of wave number 3600–3100 cm^−1^ (corresponding to O-H oscillation). In pure MC, the peak at 3501 cm^−1^ is the stretching vibration peak of the –OH bond, the strong and broad absorption peak here indicates that the hydroxyl group in MC has an association reaction. The C–H bond stretching vibration peak is at 2923 cm^−1^ and the C–O–C bond stretching vibration peak at 1082 cm^−1^. It can be seen from the spectrum that the characteristic band of pure MC at 1476 cm^−1^ disappeared, and no new characteristic band appeared. All peaks are much weaker, indicating that a large number of hydrogen bonds are formed between MC, PAAS, and MgCl_2._ The band at 3411 cm^−1^ narrows and shifts to the low-frequency range, demonstrating that a hydrogen bond can be formed between MC and PAAS. The band vibration enhancement at 1636 cm^−1^ is the result of the superposition of the C=C stretching vibration absorption peaks in MC, PAAS, and MgCl_2_ at the same place. In the spectrum of the thermosensitive hydrogel, the characteristic peaks of MC, PAAS, and MgCl_2_ are all present, indicating that there is only physical cross-linking among the three components.

### 3.2. Surface Tension Analysis

**Figure 4:**
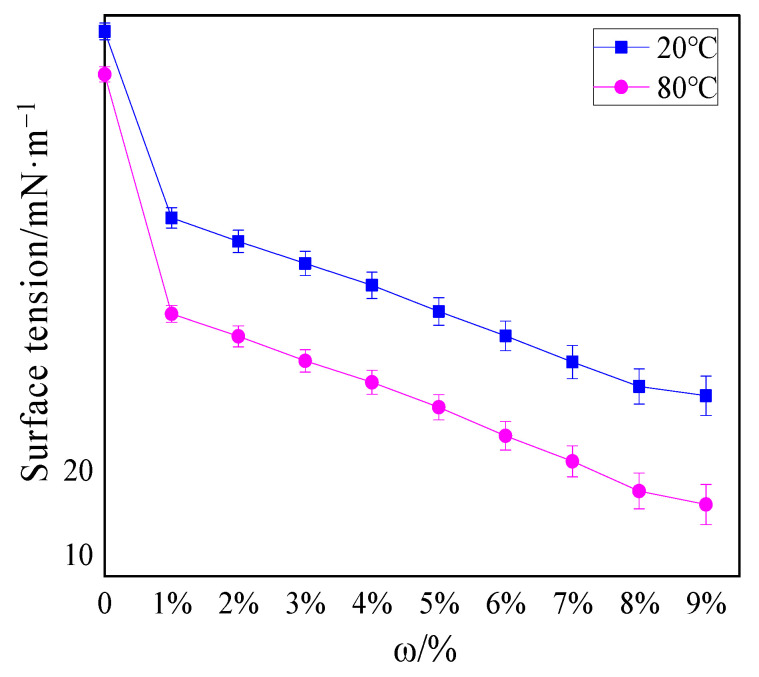
Surface tension of hydrogels with different concentrations at 20 and 80 °C.

Figure 4 shows the surface tension of thermosensitive hydrogel solutions with different concentrations at 20 and 80 °C.

The surface tension of water at 20 and 80 °C was 72.1 and 67 mN/m, respectively, while the surface tension of 1% hydrogel solution was 50 and 38.61 mN/m, respectively. As the concentrations of hydrogel increased, the surface tension decreased gradually. Significantly, the surface tension decreased to a greater extent after the temperature augmented. The surface tension of the 9% hydrogel solution at 80 °C even dropped to 16 mN/m, exhibiting temperature sensitivity. This similar phenomenon of surface tension varying with temperature was also found in Deng [22] and Jia’s [32] previous research, and the surface tension of the hydrogel prepared by them was at least 32 mN/m at 90 °C. It can be seen from Figure 4 that the decrease in surface tension of the hydrogel solution at 80 and 20 °C fluctuated between 0.55–0.83 times (all less than 0.95 times), and GB 15308- 2006 can confirm that the fire extinguishing fluid was thermosensitive extinguishing fluid.

The lower surface tension of thermosensitive hydrogels than water was the result of the interaction between hydrophilic groups and hydrophobic groups in solution, which was also the foremost reason for the thermo-sensitive characteristics of hydrogels. These results had also been explained in Deng and Jia’s research [22,32]. At 20 °C, the hydrophilic groups played a major role, and the hydrogel was fully swollen when it absorbed water. At this point, the hydrogel solution had superior surface activity; consequently, the surface tension was larger. When the temperature rose above 80 °C, the hydrophobic groups attracted each other and clung to each other, and the hydrophilic groups formed micelles outwards. Accordingly, the volume phase transition occurred in hydrogel solution, leading to weakened surface activity; consequently, the surface tension at this time was lower than that at a normal temperature. It can be inferred that when this type of thermosensitive hydrogel was employed to extinguish fires, it was easier to spread on the surface of the burning material than water, thus accelerating the absorption of hydrogel to the heat of the fire site and enhancing the extinguishing effect.

### 3.3. Viscosity Analysis

**Figure 5:**
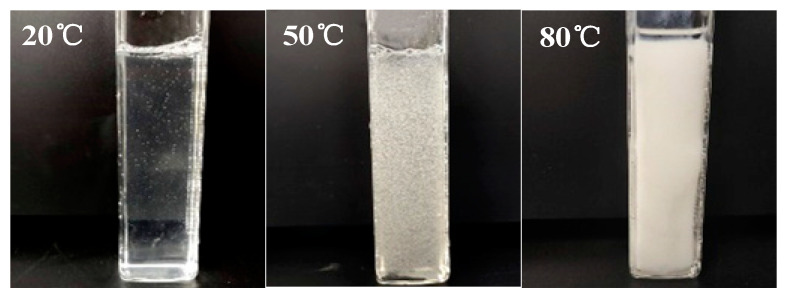
The phase change of thermosensitive hydrogels at different temperatures.

The thermosensitive hydrogel undergoes significant phase changes under various temperature conditions, the typical process is shown in Figure 5.

Figure 5 displays that the thermosensitive hydrogel remains sol at 20 °C and gel at 80 °C. This is because part of the hydroxyl groups of the hydrogel molecular chain forms intermolecular hydrogen bonds at low temperatures, resulting in chain entanglement. As the temperature elevated, molecular thermal motion intensified and the hydrogen bonds between molecular chains were destroyed gradually [33]. The increase in temperature promoted the hydrophobic interaction between the hydrophobic groups of the molecular chain, resulting in the formation of the gel. 

**Figure 6:**
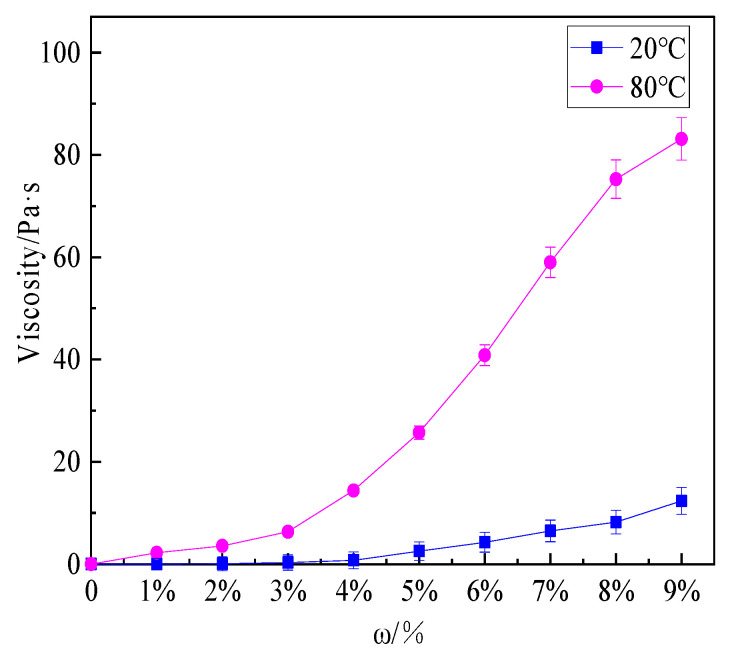
Viscosity of hydrogel solutions with different mass fractions at 20 °C and 80 °C.

Figure 6 shows that the viscosity of the hydrogel solutions increased with the increase in concentration and temperature. 

The viscosity of the hydrogel with a concentration of 9 wt% at 20 and 80 °C was 12.35 Pa·s and 83.12 Pa·s, respectively. At 20 °C, the viscosity of the hydrogel solution increased slightly. After heating to 80 °C, the phase of hydrogel solution changed from sol to gel, the fluidity decreased and the viscosity increased. As the concentration increases, the viscosity of the hydrogel solution considerably increased. The viscosity of the thermosensitive hydrogel prepared by Deng and Jia also increased by 4–5 times or even 8–9 times under the conditions of 20 °C and 90 °C [22,34]. The enhanced viscosity was conducive to the adhesion of the gel to the surface of the combustion material, distinctly improving the cooling and sealing effect of the extinguishing agent. In this paper, four hydrogel solutions with different concentrations of 2 wt%, 4 wt%, 6 wt%, and 8 wt% were selected for small-scale fire extinguishing experiments, and then a hydrogel solution suitable for actual fire extinguishing was selected.

### 3.4. Small-Scale Fire Extinguishing Performance

**Figure 7:**
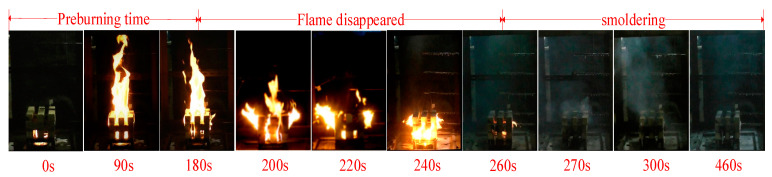
Flame morphology of thermosensitive hydrogel of 6wt% during the fire suppression process.

Small-scale fire extinguishing tests of four samples (different concentrations of 2 wt%, 4 wt%, 6 wt% and 8 wt%) were conducted to assess the fire extinguishing performance of a thermosensitive hydrogel. Owing to thermosensitive hydrogels with different concentrations having similar morphology during the fire suppression process, the morphology of the fire suppression process with 6wt% of the hydrogel is shown in Figure 7.

**Figure 8:**
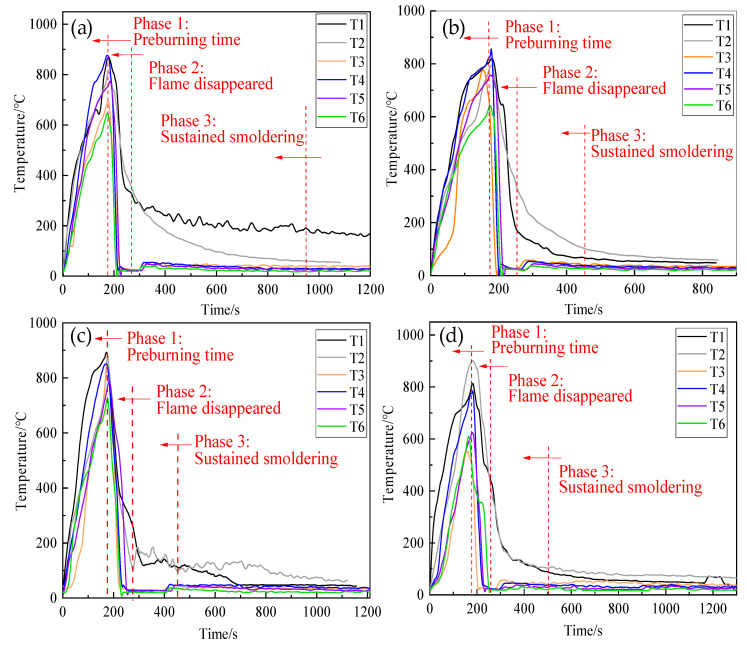
Variation of flame temperature in the tests with four samples as suppressants: (**a**) 2 wt% thermosensitive hydrogel; (**b**) 4 wt% thermosensitive hydrogel; (**c**) 6 wt% thermosensitive hydrogel; (**d**) 8 wt% thermosensitive hydrogel.

Variation of flame temperature for the four samples was achieved by six thermocouples, as shown in Figure 8. Compared to thermosensitive hydrogel with concentrations of 8 wt%, the temperatures in Figure 7a,b decreased relatively quickly, demonstrating thermosensitive hydrogel with concentrations of 2 wt%,4 wt% can extinguish open fire in a relatively fast time. 

Table 1 lists the fire extinction time. Owing to their having a lower concentration and stronger fluidity, they flowed to the center and bottom of the wood stack at a relatively faster rate after spraying. However, their gel strengths were lower after phase transformation, and they maintained a long duration of smoldering of 580 and 320 s, respectively. Therefore, their total fire-extinguishing time was longer. While the thermosensitive hydrogel with concentrations of 6 wt% and 8 wt% had a relatively longer extinguishing time, their ability to suppress smoldering was prominently improved; the total fire-extinguishing time of the thermosensitive hydrogel with concentrations of 6 wt% and 8 wt% was relatively short at 280 and 295 s, respectively, and no re-ignition phenomena were observed. The above results revealed that thermosensitive hydrogel with concentrations of 6 wt% exhibited much better fire performance in terms of fire extinguishing time.

Therefore, the thermosensitive hydrogel with a concentration of 6 wt% was employed for a large-scale fire extinguishing experiment with ordinary gel and foam extinguishing agents, which were uniformly diluted to 6 wt% to compare the fire extinguishing performance of these three types of fire extinguishing agents. 

### 3.5. Large-Scale Fire Extinguishing Experiment Results

#### 3.5.1. Fire Extinguishing Performance

**Figure 9:**
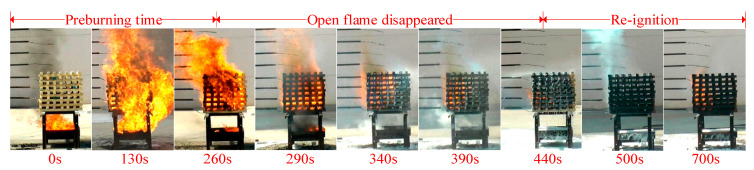
Fire extinguishing process images: foam extinguishing agent.

Figure 9 illustrates the flame morphology of foam extinguishing agent during the fire extinguishing process. The extinguishing process of three kinds of extinguishing agents can be divided into three stages, including the pre-burning stage (0–260 s), the open fire extinguishing stage, and the re-ignition stage. In the pre-burning stage, the T1 thermocouple placed at the bottom of the wood stack first heated up when the oil fire in the ignition plate was ignited, and the maximum temperature of the thermocouple was above 900 °C.

**Figure 10:**
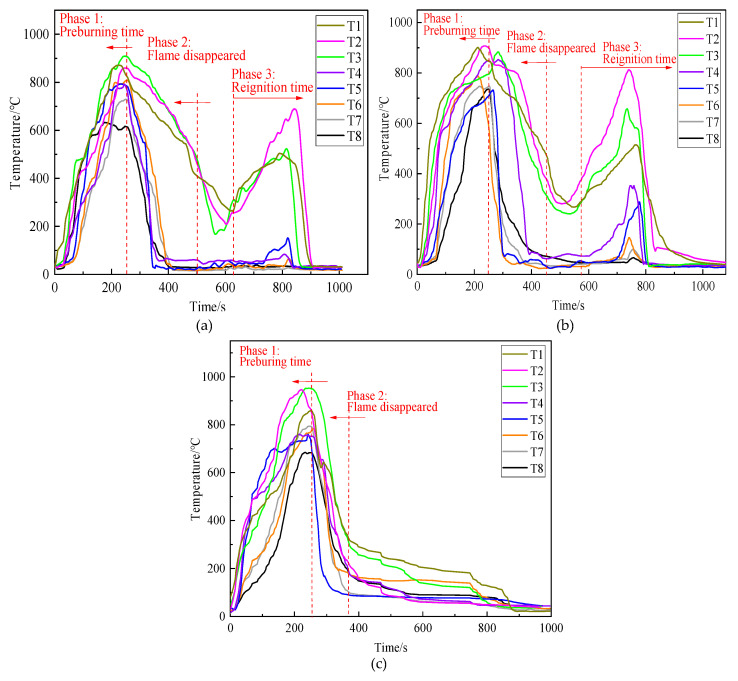
Flame temperature curves of: (**a**) ordinary gel extinguishing agent, (**b**) foam extinguishing agent, (**c**) 6 wt% thermosensitive hydrogel.

Figure 10 shows the variation in the temperature during fire extinction experiments. When equivalent volumes of the three kinds of extinguishing agents were used separately for fire extinction, the four thermocouples located above the wood stack, namely T1, T2, T3, and T4, showed a significant decrease in temperature. Owing to the wood stack fire being a deep fire, T5–T8, which were located inside the wood stack, showed a slower decrease in temperature. Even the thermocouples of T5–T8 in Figure 9a,b dropped to 620 s and 580 s, respectively; the temperature rose again, indicating that ordinary gel and foam extinguishing agents had reignited during the fire extinguishing process. Figure 9c pictured the flame temperature curve of the thermosensitive hydrogel, recording a faster decrease in temperature than Figure 9a,b, and no re-ignition phenomena were observed after the open fire disappeared. 

Table 2 lists the performance parameters and fire extinction time of three samples. They can extinguish the open flame by 240, 180, and 120 s, respectively. However, re-ignition phenomena occurred in Figure 9a,b. The total fire-extinguishing times of ordinary gel and the foam extinguishing agent are 340 and 240 s, respectively. Comparatively, the total fire-extinguishing time of thermosensitive hydrogel was short at 120 s, and no re-ignition occurred. The results revealed that thermosensitive hydrogel exhibited better fire extinguishing performance than ordinary gel and foam extinguishing agents.

#### 3.5.2. Cooling Performance 

The cooling effect is an important fire extinguishing mechanism of a water-based fire extinguishing agent. The water deposited on the surface of the burning material can absorb a great deal of heat. Simultaneously, the water film or foam layer formed on the surface of the burning material will act as a heat insulation barrier to insulate oxygen. 

Due to the fire extinguishing agents being sprayed on the wood stack after 260 s of pre-combustion in these experiments. In this study, eight coordinate points were selected in each temperature curve ranging from 260 s to 400 s for linear fitting, respectively, to test the cooling efficiency of the three types of fire extinguishing agents. The fitting line slope was the cooling rate of the test points. The slope average of the eight test points can be regarded as the average cooling rate. Linear fitting results are shown in Table 3.

It can be seen from Table 3 that the maximum cooling rate of ordinary gel, the foam extinguishing agent and thermosensitive hydrogel are 7.22, 6.39 and 8.77 °C/s, respectively. Moreover, the average cooling rates of these three extinguishing agents are 3.92, 3.68 and 5.19 °C/s, respectively. The test results showed that the cooling efficiency of the 6 wt% thermosensitive hydrogel is better than the ordinary gel and foam extinguishing agents.

#### 3.5.3. Fire Extinguishing Mechanism

According to the combustion theory, the fire extinguishing mechanism of fire extinguishing agents is divided into four types: cooling, isolation, suffocation and suppression. In previous studies, Jia [34] discovered that P(NIPA/AA-Na) thermosensitive hydrogel had a shorter extinguishing time and faster cooling ability than gel B in the process of extinguishing oil pan fire, and he concluded this was caused by the foam-like gel produced by the thermosensitive hydrogel at high temperature. Yang [35] conducted Class 1A wood stack fires and extinguished them using water, ordinary gel and thermosensitive hydrogel P (NIPA-co-SA), respectively; she concluded that the performances of cooling and extinguishing of thermosensitive hydrogel were better than water and ordinary gel. However, the thermosensitive hydrogels prepared by them were almost chemically synthetic hydrogels, which were difficult use in actual production and fire extinguishing due to the relatively complex synthesis process and relatively high cost.

In our study, a thermosensitive hydrogel was synthesized by physical mixing of methyl cellulose, sodium polyacrylate and magnesium chloride. In the process of fire extinguishing, the flame temperature outside the wood stack was as high as 900 °C when the wood stack fire was completely burned, which was much higher than the LCST required for the phase change of the thermosensitive hydrogel; the phase transition occurred rapidly after the hydrogel reached the combustion zone, and the viscosity grew rapidly. The gel adhered to the surface of the wood stack, reducing the contact area between the wood stack and the air, playing a role in isolating the surrounding oxygen and slowing down the oxidative heat release of the wood stack; simultaneously, it had the effect of cooling, suffocating and blocking, which enhanced the use efficiency of fire extinguishing agents [34,35]. Furthermore, the other two substances also played an important role in extinguishing the fire. PAAS formed a fire-resistant layer after absorbing water, thus playing a role in resisting flames [29], MgCl_2_ had catalytic action on the polymerization of levoglucosan in cellulose at 250 °C and increased the primary char yield of cellulose heated at 400 °C, thereby effectively improving the fire resistance of the temperature-sensitive hydrogel [30,31]. Therefore, the fire extinguishing performance and the anti-re-ignition performance of thermosensitive hydrogel were extensively enhanced.

## 4. Conclusions

In this study, a type of thermosensitive gel additive was prepared by compounding methylcellulose, sodium polyacrylate, and magnesium chloride. The functional groups were characterized, the surface tension and viscosity of the thermosensitive hydrogel solutions with different concentrations before and after the phase change were analyzed, and small-scale and large-scale experiments were carried out to test the fire extinguishing performance, and the following conclusions were obtained:

The thermosensitive hydrogel has a lower viscosity and stronger fluidity at 20 °C. The phase transition occurred when the temperature was higher than 80 °C, the viscosity was notably boosted, and the surface tension was reduced. It can be inferred that when it is used as a water-based fire extinguishing agent additive, it can enhance adhesion and spreading performance on the surface of the burning material, which is beneficial to improve the fire extinguishing efficiency. The fire extinguishing efficiency of different concentrations of thermosensitive hydrogels were compared through small-scale fire extinguishing experiments, and then a thermosensitive hydrogel solution with a concentration of 6 wt% was selected. The fire-extinguishing time is 80 s and the total fire-extinguishing time is 280 s.

In order to further verify the fire extinguishing performance of thermosensitive hydrogel, the large-scale fire platform was designed according to GB 17835-2008. Fire extinguishing experiments were carried out to compare the fire extinguishing efficiency of 6 wt% thermosensitive hydrogel and ordinary gel and foam extinguishing agents. The results showed that the extinguishing performance of thermosensitive hydrogel was better than the other two other fire extinguishing agents from the analysis of extinguishing time and cooling efficiency. It has great application prospects.

## Figures and Tables

**Table 1:** Fire extinguishing performance of the four samples.

Concentration of Extinguishing Agent/wt%	Extinguishing Time/s	Smoldering Time/s	Total Fire-Extinguishing Time/s
2	70	580	650
4	74	320	404
6	80	200	280
8	115	180	296

**Table 2:** Performance parameters and fire extinguishing time of three samples.

Types	20 °C		80 °C		Extinguishing Open Flame Time/s	Total Fire Extinguishing Time/s
	Surface Tension/mN·m^−1^	Viscosity/Pa·s	Surface Tension/mN·m^−1^	Viscosity/Pa·s		
Ordinary gel extinguishing agent	26.4	28.4	25.1	28.1	240	340
Foam extinguishing agent	20.6	0.068	19.8	0.054	180	240
Thermosensitive hydrogel	36	4.256	24.15	40.858	120	120

**Table 3:** The cooling efficiency linear fitting results of extinguishing agents.

Curve	Fitting Equation		R^2^	V_max_ (°C/s)	V_aver_ (°C/s)
Ordinary gel extinguishing agent	T1	−1.35t + 1149.30	0.993	7.22	3.92
	T2	−1.34t + 1211.27	0.971		
	T3	−1.71t + 1368.59	0.970		
	T4	−5.19t + 2048.99	0.961		
	T5	−7.22t + 2711.39	0.904		
	T6	−5.55t + 2308.62	0.962		
	T7	−4.46t + 1853.51	0.987		
	T8	−4.55t + 1768.50	0.968		
Foam extinguishing agent	T1	−1.67t + 1250.49	0.980	6.39	3.68
	T2	−2.11t + 1458.21	0.809		
	T3	−2.85t + 1640.42	0.837		
	T4	−6.39t + 2669.48	0.878		
	T5	−5.52t + 2027.79	0.673		
	T6	−3.11t + 1180.40	0.663		
	T7	−4.14t + 1588.64	0.782		
	T8	−3.67t + 1502.39	0.841		
Thermosensitive hydrogel	T1	−4.09t + 1830.95	0.944	8.77	5.19
	T2	−5.03t + 2080.56	0.951		
	T3	−5.49 + 2353.12	0.943		
	T4	−4.59t + 1917.28	0.937		
	T5	−8.77t + 2833.85	0.805		
	T6	−5.15t + 2030.13	0.800		
	T7	−4.64t + 1849.30	0.859		
	T8	−3.76t + 1585.72	0.917		

## Data Availability

The data presented in this study are available on request from the corresponding author. The data are not publicly available due to the data required to reproduce these findings forms part of an ongoing study.
